# Supercontinuum generation in dispersion engineered AlGaAs-on-insulator waveguides

**DOI:** 10.1038/s41598-021-81555-3

**Published:** 2021-01-21

**Authors:** Stuart May, Matteo Clerici, Marc Sorel

**Affiliations:** 1 James Watt School of Engineering, University of Glasgow, Glasgow, G12 8QQ UK; 2 Institute of Technologies for Communication, Information and Perception (TeCIP), Sant’Anna School of Advanced Studies, Via Moruzzi 1, 56127 Pisa, Italy

**Keywords:** Integrated optics, Optics and photonics, Nonlinear optics, Supercontinuum generation, Nanoscience and technology, Nanoscale devices

## Abstract

The effect of engineering the dispersion of AlGaAs-on-insulator (AlGaAs-OI) waveguides on supercontinuum generation is investigated at telecom wavelengths. The pronounced effect the waveguide width has on the nonlinear dynamics governing the supercontinua is systematically analyzed and the coherence of the spectra verified with numerical simulations. Using dispersion engineered AlGaAs-OI waveguides, broadband supercontinua were readily obtained for pulse energies of $\sim \text {3 pJ}$ and a device length of only 3 mm. The results presented here, further understanding of the design and fabrication of this novel platform and describe the soliton and dispersive wave dynamics responsible for supercontinuum generation. This study showcases the potential of AlGaAs-OI for exploring fundamental physics and realizing highly efficient, compact, nonlinear devices.

## Introduction

Ever since the invention of the GaAs laser in 1962^1^, the GaAs/AlGaAs material platform has been of significant importance for integrated optics. It is of particular interest as the high nonlinearity of the material make it ideal for nonlinear optical processing, where the femtosecond response time can be utilized for processing signals at speeds beyond a terabit-per-second in compact, power efficient devices^2^. AlGaAs also offers the ability to engineer its bandgap by varying the composition of the alloy, thus providing a degree of freedom in device design. This property has been widely exploited to develop waveguides that do not suffer from two photon absorption (TPA) at telecommunication wavelengths^3,4^. These numerous benefits, combined with a wide transparency window ($\sim 0.9{-}17\mu \text {m}$ for GaAs^5^), make for a very versatile platform and because of that GaAs/AlGaAs waveguides have been employed in a wide range of applications such as quantum optics^6^, molecular sensing^7^ and telecommunications^8^. Despite the maturity and success of this platform, the low refractive index contrast between AlGaAs layers means that high aspect ratio, sub-micron scale waveguides are often needed to satisfy the phase matching conditions or dispersion profiles required for nonlinear processes. Such waveguides can be challenging to fabricate, frequently resulting in nonlinear devices with high propagation losses^4,9^.

In 2015 the heterogeneous integration of AlGaAs-on-insulator (AlGaAs-OI) was found to be an elegant solution to overcome the limitations of GaAs/AlGaAs waveguides^10^. When AlGaAs is bonded to a silica cladding layer, there is a significant increase in the vertical modal confinement ($\Delta \text {n}\approx \text {1.82}$), which enables the fabrication of sub-micron scale waveguides with effective nonlinearities as high as $\text {720}~{\text {W}}^{-1}{\text {m}}^{-1}$^11^. This high nonlinearity together with low loss waveguides has allowed for the fabrication of high Q factor microring resonators, making Kerr frequency comb generation possible for microwatt levels of input power^12^. In addition to Kerr nonlinearities, the non-centrosymmetric structure of AlGaAs enables highly efficient second harmonic generation in this platform^13,14^.

In this work, we report a systematic analysis of the effect that dispersion engineering has on the nonlinear dynamics governing supercontinuum generation (SCG) in an AlGaAs-OI waveguide. Specifically, we explore how the width of the waveguide can be utilised to dispersion engineer the waveguide for efficient, broadband SCG. By varying the dimensions of the waveguide, we demonstrate that the dispersion at the pump wavelength can be tailored to enable us to investigate different dispersive regimes in the vicinity of two zero dispersion wavelengths. These regimes lead to notable changes in the nonlinear dynamics that govern the spectral broadening. Supercontinuum generation is an important nonlinear process, which finds applications in a number of fields including metrology, optical coherence tomography, spectroscopy and pulse compression^15^. It has also been instrumental for investigating exotic phenomena such as rogue waves^16^ and dark soliton interactions^17^. This work is therefore crucial for the development of the next generation of compact, power efficient supercontinuum (SC) sources and enhances our understanding of the design and implementation capabilities of the AlGaAs-OI platform.

## Design

When dispersion engineering a waveguide for broadband SCG, it is beneficial to have a group velocity dispersion (GVD = $\frac{\partial ^{2}\beta }{\partial \omega ^{2}}$; for propagation constant $\beta$, and frequency $\omega$) profile which is low and flat over a wide spectral band. Low dispersion minimizes the temporal walk-off during spectral broadening, thus maximizing the interaction length that is required for efficient nonlinear effects and phase matching conditions^18^. By having a flat dispersion profile, third order dispersion (TOD = $\frac{\partial ^{3}\beta }{\partial \omega ^{3}}$) is suppressed which is advantageous for supercontinua based on broadening via nonsolitonic radiation or modulation instability^19,20^. As such, designs for SCG predominantly aim for the pump wavelength to be in the vicinity of the zero dispersion point^21^.

**Figure 1:**
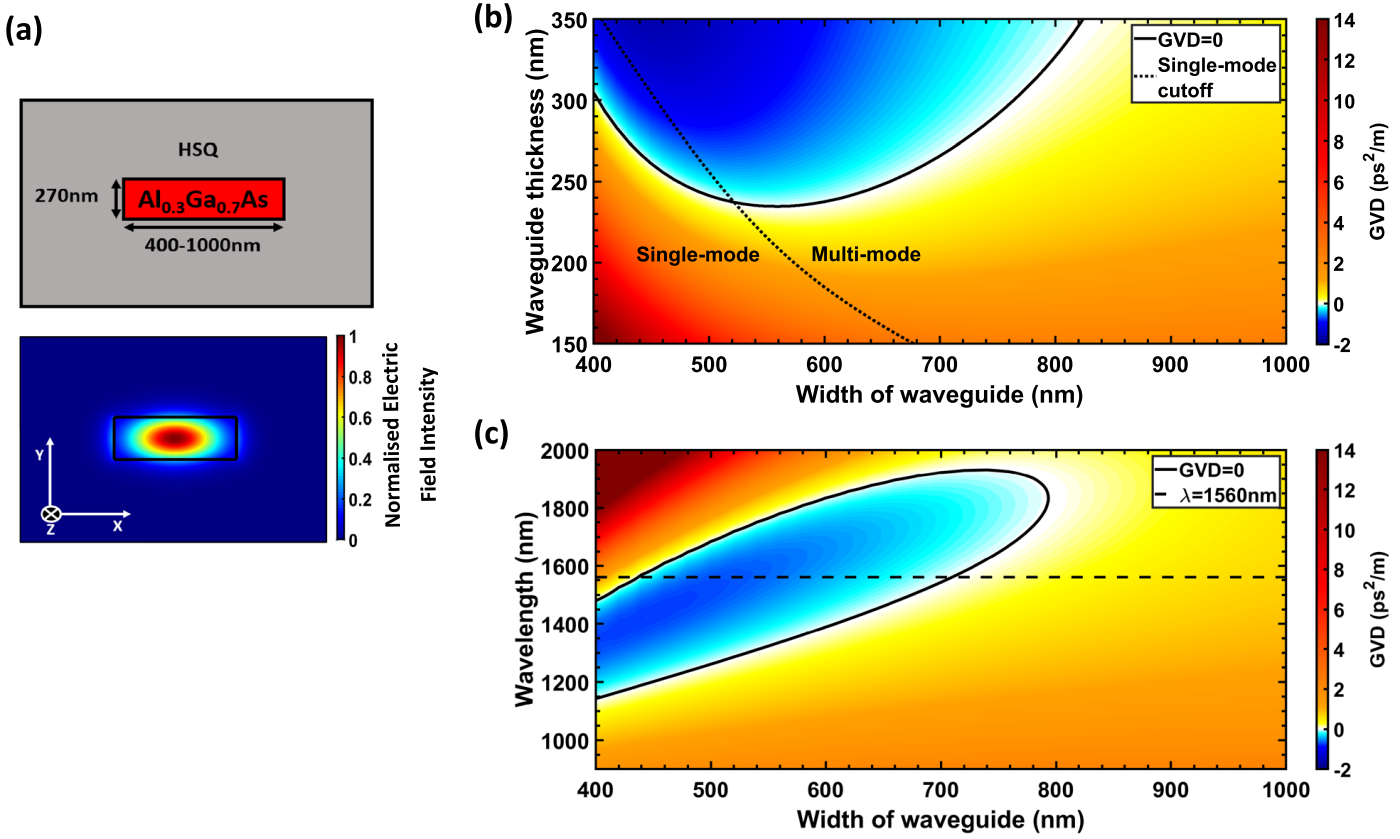
Dispersion engineering of AlGaAs-OI waveguides. (**a**) Diagram of the waveguide cross section and corresponding electric field intensity of the fundamental quasi-TE mode used for SCG, where z is the propagation axis into the page ($\lambda =1560 \, \text {nm}$). (**b**) Simulation showing how the group velocity dispersion, GVD, of the fundamental quasi-TE mode at a fixed wavelength of 1560 nm varies with both the width and thickness of the ${\text {Al}}_{0.3}{\text {Ga}}_{0.7}\text {As}$-OI waveguide. The solid black line highlights the dimensions where the GVD is equal to zero whilst the dashed black line indicates where the waveguide becomes multi-mode i.e. when higher order modes begin to propagate. (**c**) Simulation depicting the GVD against width and wavelength for a fixed waveguide thickness of 270nm. Zero GVD and the pump wavelength at 1560nm are shown with a solid and dashed black lines, respectively. All simulations were conducted using the finite difference eigenmode (FDE) solver from Lumerical, Inc.

A common method to obtain the desired dispersion for SCG is to tailor the dimensions of the waveguide. Simply by varying the geometry of the waveguide, one can compensate for the strong normal dispersion of bulk materials with the waveguide dispersion^22^. As shown in Fig. 1b, by altering both the thickness and width of the ${\text {Al}}_{0.3}{\text {Ga}}_{0.7}\text {As}$-OI waveguide, the total dispersion of the fundamental quasi-TE mode—Fig. 1a can be designed to be either anomalous ($\hbox {GVD} < 0$) or normal ($\hbox {GVD} > 0$) at the pump wavelength ($\lambda$ = 1560 nm). In this case an aluminum percentage of 30% was chosen for the AlGaAs alloy (${\text {E}}_{g}$  = 1.8 eV) to avoid TPA at the pump wavelength and hydrogen silsesquioxane (HSQ) used as an upper and lower cladding for the waveguide. When thermally annealed, HSQ forms a ${\text {SiO}}_{2}$-like structure with a refractive index $\text {n}\sim 1.39$ at 1560 nm (verified with ellipsometry)^23^.

To examine the effect different dispersion regimes have on SCG, a thickness of 270 nm was considered for this study. This thickness offers two zero-dispersion wavelengths (ZDWs) in the vicinity of the pump, i.e. wavelengths where GVD=0 - see Fig. 1c, which allows for the efficient generation of dispersive waves (DWs). Anomalous dispersion can also be obtained whilst remaining in the single-mode regime for this thickness, see Fig. 1b, which is advantageous for generating SCG with high spatial coherence.

### Fabrication

**Figure 2:**
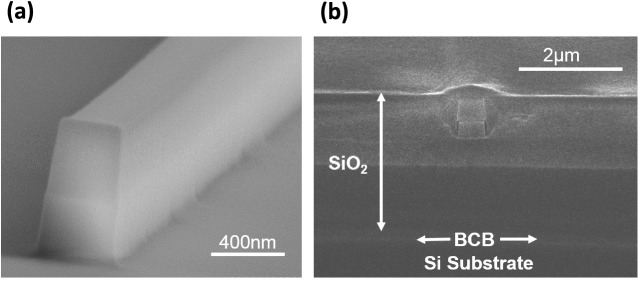
AlGaAs-OI waveguides. (**a**) Scanning electron microscope (SEM) image of an etched AlGaAs-OI waveguide showing minimal sidewall roughness. (**b**) SEM image showing the cross section of the final AlGaAs-OI device.

Metal organic chemical vapor deposition was used to epitaxially grow 270 nm of ${\text {Al}}_{0.3}{\text {Ga}}_{0.7}\text {As}$ atop lattice matched InGaP etch stop layers on a GaAs substrate. A layer of HSQ (Dow Corning FOX-15) was then deposited on the wafer, followed by $3~\upmu \text {m}$ of plasma enhanced chemical vapor deposition silica. This combined layer formed the buried oxide (BOX) layer of the final device. Adhesive sample bonding with benzocyclobutane (BCB) was subsequently employed to bond the GaAs/AlGaAs sample to a host silicon substrate. During the bonding process, constant pressure was applied to the material stack whilst the BCB was cured at 250 °C on a hotplate. After successful bonding of the samples, in order to form the final AlGaAs-OI material platform, both the GaAs substrate and InGaP etch stops of the GaAs/AlGaAs were removed using citric acid/hydrogen peroxide ($4:1$ volumetric ratio) and HCl acid, respectively. Using electron beam lithography, a HSQ hard mask was then defined and a ${\text {SiCl}}_{4}/\text {Ar}/{\text {N}}_{2}$ inductively coupled plasma dry etch used to transfer the waveguide pattern to the AlGaAs layer below with minimal sidewall roughness—Fig. 2a. Finally, the waveguides were cladded in HSQ and cleaved for end fire coupling—Fig. 2b.

## Supercontinuum generation

**Figure 3:**
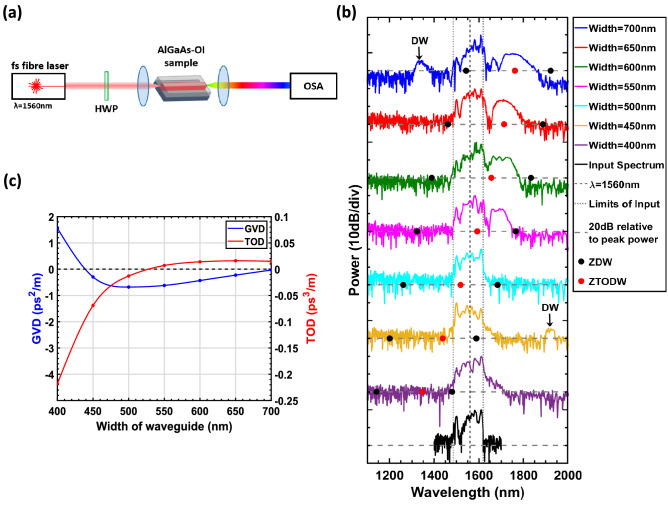
Supercontinuum spectra (**a**) Experimental setup used for SCG (**b**) Experimental results of how the SC spectra vary depending on the waveguide width. For each waveguide width, the pulse energy in the waveguide was $\sim\,3.1$ pJ. The zero dispersion wavelength (ZDW) and zero third-order dispersion wavelengths (ZTODW) i.e. where the third order dispersion (TOD) equals zero, are also shown for each waveguide and, where applicable, the dispersive wave (DW) is indicated. (**c**) Simulation showing how the group velocity dispersion (GVD) and third order dispersion at the pump wavelength ($\lambda$ = 1560 nm) change with the width of the waveguide.

To characterize the AlGaAs-OI waveguides for SCG, a laser source providing pulses with 100 fs duration at a repetition rate of 80 MHz and a center wavelength of 1560 nm was used. This laser was polarized and the polarization was controlled with a half wave plate (HWP) before it was end fire coupled in and out of the AlGaAs-OI waveguide by 40x (NA = 0.65) microscope objectives and coupled to an optical spectrum analyzer (OSA)—see Fig. 3a. Since spot size converters were not implemented in the waveguide design, there was a large modal mismatch (~ 9 dB when also considering Fresnel reflections) when light was coupled to and from the waveguides resulting in a measured average coupling loss of $\sim 12$ dB/facet.This also meant that the generated SC in the multi-mode waveguides were sensitive to the input coupling position. For this experiment, all waveguides had a length of 3 mm and propagation losses were measured via the Fabry-Pérot loss measurement technique^24^ to be 2–3 dB/cm for the fundamental TE mode.

Using this setup, the results shown in Fig. 3b were obtained. The SC was measured for waveguide widths varying from 400 to 700 nm for a fixed energy of approximately 3 pJ coupled to the waveguide.

For a waveguide width of 400 nm, the pump wavelength lies within the normal dispersion regime meaning the broadening of the input is mainly attributed to self-phase modulation (SPM). Since SPM is a self-seeded process, this results in a smooth and stable output spectrum^25^. The large normal dispersion at the pump wavelength—Fig. 3c—however, means that the pulse rapidly disperses before any significant broadening can occur.

As the waveguide width is increased to 450 nm, the majority of the pump is within the anomalous dispersion regime. Since the pump is in close to the ZDW, efficient energy exchange occurs between a generated soliton and a DW^20^. Due to the negative TOD at the pump, the DW is emitted on the red side of the soliton at a center wavelength of 1922 nm, as dictated by the phase matching condition for Cherenkov radiation^21,26^. Bright solitons cannot exist in the normal dispersion regime, therefore broadening of the pump for wavelengths greater than the ZDW occurs due to SPM.

For a waveguide width of 500 nm, the GVD at the pump is the most negative out of all the waveguides considered. This high anomalous dispersion means that the phase matching condition for DW formation is no longer satisfied. More energy is also required to support the fundamental soliton, thus less energy is available to broaden the pulse. Furthermore, negative TOD at the pump suppresses any soliton self-frequency shift (SSFS)^26,27^. A combination of these factors results in minimal broadening of the pulse.

When the waveguide width is between 550 and 650 nm, the GVD at the pump is anomalous and tends towards zero as the width increases across this range. At the same time, the TOD becomes increasingly more positive and soliton dynamics are the dominant phenomena responsible for SCG^25^. In this case the supercontinua are able to span over the majority of the anomalous dispersion regime, because nonlinearities are enhanced for low values of GVD and large TOD helps to break up higher order solitons and increase the growth rate of SSFS^26^.

**Figure 4:**
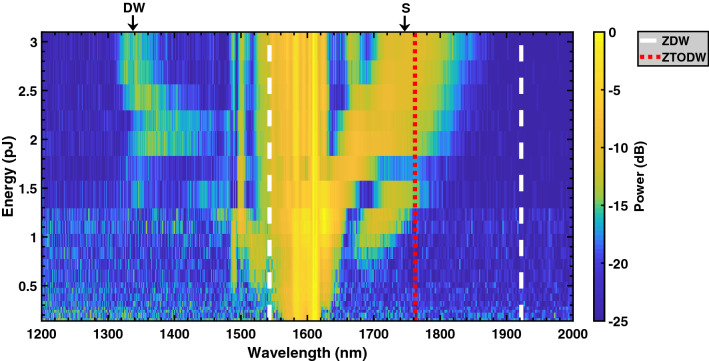
Evolution of the generated supercontinuum of a 700 nm wide AlGaAs-OI waveguide as the energy coupled to the waveguide is increased. The dashed line shows the zero dispersion wavelengths (ZDWs), whilst the dotted line displays the zero third-order dispersion wavelength (ZTODW). A soliton (S) and dispersive wave (DW) pair have also been indicated.

For this experiment the largest SC, spanning ~ 544 nm (at − 25 dB level), was obtained for a waveguide width of 700 nm—Fig. 3b. In this case, the pump wavelength is in the vicinity of a ZDW with positive TOD meaning a dispersive wave is emitted on the blue side of the soliton at a center wavelength of 1335 nm^20,26^. On the red side of the input, solitons help broaden the signal towards the second ZDW. To better understand the phenomena responsible for this broadband SC, the output spectra of the waveguide was measured as the input pulse energy was increased (see Fig. 4). For input energies $\lesssim$ 0.7 pJ, SPM is the dominant mechanism responsible for the symmetric broadening of the pulse. Above this energy, fission of the input pulse occurs resulting in the formation of solitons and dispersive waves. As the energy is increased further, SSFS is enhanced thus corresponding to a greater red-shift of the generated solitons. Since a soliton is coupled to a dispersive wave, spectral recoil induces a further red-shift of the soliton whilst simultaneously shifting the DW towards shorter wavelengths^28^. Above $\gtrsim$ 2.5 pJ, a saturation in this shift is observed owing to the suppression of SSFM from negative TOD at the soliton and its proximity to the second ZDW^20^. Three photon absorption (3PA) and surface enhanced third harmonic generation^29^ at the input facet were also noted for high input powers which will also constrict the broadening of the SC^30,31^.

Both Figs. 3 and 4 demonstrate the successful dispersion engineering of an AlGaAs-OI waveguide and the marked effect it has on the observed nonlinear phenomena. Thanks to the superior nonlinear properties of AlGaAs, broadband SC spectra were readily obtained in a device of only 3 mm in length and for pulse energies as low as ~ 2.5 pJ. These results illustrate the ability of the platform for realizing compact, power efficient PICs for nonlinear optics.

## Discussion

When using SCG for applications in frequency metrology, frequency comb generation and optical coherence tomography, a high degree of temporal coherence is required. To verify the coherence of the generated spectra, the modulus of the first order coherence, $|g_{12}^{(1)}|$, was calculated as:

1 $$\begin{aligned} \big |g_{12}^{(1)}(\lambda )\big |=\Bigg |\frac{\langle E^{*}_{1}(\lambda )E_{2} (\lambda )\rangle }{\sqrt{\langle |E_{1}(\lambda )|^{2}\rangle \langle |E_{2}(\lambda )|^{2}\rangle }}\Bigg | \end{aligned}$$

where E1 and E2 are individual SC spectra numerically simulated by solving the general nonlinear Schrödinger equation (GNLSE)^21,32^. The coherence in our case was taken by considering the ensemble average of 500 individually computed spectra, where quantum noise was modeled as one photon per mode noise and an intensity noise of 1.5% assumed for the input pulse condition, following the procedure proposed in^33^.

**Figure 5:**
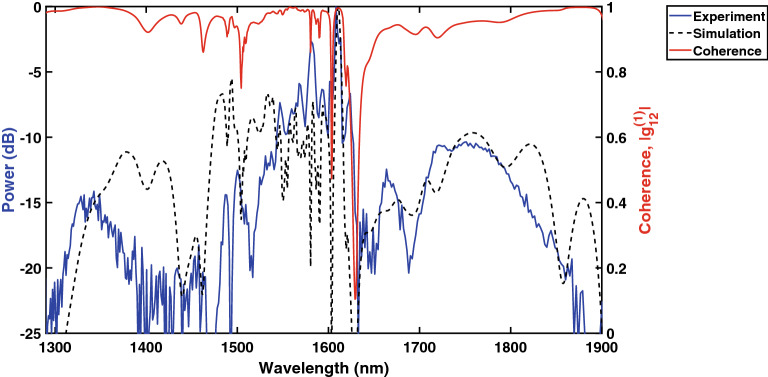
Experimental and simulated spectra (left y-axis) of SCG in a 700nm wide AlGaAs-OI waveguide for an input energy of 3.1pJ. The calculated coherence is also shown for this waveguide (right y-axis).

As shown in Fig. 5, good agreement between simulation and experiment was obtained and a high degree of coherence calculated for the SC generated from the 700 nm wide AlGaAs-OI waveguide. Small discrepancies between the simulated and experimental spectra can be expected owing to the sensitivity of the dispersion to fabrication tolerances.

To further improve the broadening of the spectra obtained, there are numerous approaches which can be explored. For example, currently a decrease in the confinement factor as the mode tends towards cut-off is the main factor limiting broadening towards longer wavelengths. This is because as the mode approaches cut-off, it also coincides with a decrease in the nonlinearity of the waveguide and an increase in both the propagation loss and dispersion of the mode. As for expanding the SC towards the blue, surface state absorption can be significant, thus resulting in an increase in propagation loss^34^. With larger waveguides and surface passivation both of these constraints can be alleviated^35^. Simply increasing the thickness of the waveguide allows for the generation of a DW at both short and long wavelengths making octave spanning SC achievable in an AlGaAs-OI platform^31^. Thanks to the high nonlinearity of the AlGaAs-OI platform, octave spanning SC can be obtained for lower pulse energies than what is required in other material platforms such as silicon^36^, silicon nitride^37^ and aluminium nitride^38^.

In future devices, the incorporation of tapers^39^, higher order modes^40^, and choice of upper cladding^41^ could also be investigated for dispersion engineering purposes thus providing a multitude of new design parameters to expand and optimize SCG.The incorporation of inverse taper couplers could also improve the coupling efficiency of the device and avoid the possible excitation of higher order modes, whilst microring resonators could be used to enhance observed nonlinearities. Moreover the strong $\chi ^{(2)}$ of AlGaAs and its corresponding second harmonic signal, lends themselves to applications requiring f-2f referencing^42^ and highlights the potential of AlGaAs-OI for examining the interplay of second and third order nonlinearities within a single material platform.

## Conclusions

We demonstrated the successful dispersion engineering of an AlGaAs-OI waveguide for supercontinuum generation by varying the width of the waveguide, and systematically analyzed the pronounced effect this had on the observed nonlinear behavior. Due to the high nonlinearity of the material platform broadband, SCG was obtained in a compact device of only 3 mm in length and for pulse energies of ~ 3 pJ. These results highlight the potential of AlGaAs-OI for power efficient photonics and for applications in metrology and optical coherence tomography. This work furthers understanding of this novel platform and illustrates its potential for investigating a plethora of nonlinear phenomena.

## Data Availability

All relevant data present in this publication can be accessed at: 10.5525/gla.researchdata.1105.
